# Validation of SOFA-2 score in sepsis and exploration of its extension with additional immune markers

**DOI:** 10.1016/j.jointm.2025.12.003

**Published:** 2026-01-08

**Authors:** Fei Pei, Bin Gu, Zimeng Liu, Guangzhen Li, Yao Nie, Minying Chen, Yongjun Liu, Xiangdong Guan, Qingui Chen, Jianfeng Wu

**Affiliations:** 1 Department of Critical Care Medicine, The First Affiliated Hospital, Sun Yat-sen University, Guangzhou, Guangdong, China; 2 Guangdong Clinical Research Center for Critical Care Medicine, Guangzhou, Guangdong, China; 3 Clinical Trials Unit, The First Affiliated Hospital, Sun Yat-sen University, Guangzhou, Guangdong, China

**Keywords:** Sepsis, Sequential organ failure assessment, Validation study, Immune system, Biomarkers

## Abstract

**Background:**

The Sequential Organ Failure Assessment (SOFA) score is central to the diagnosis and management of sepsis, but its recent update has not been specifically evaluated in sepsis. Moreover, it is unknown whether adding immune markers beyond white blood cell (WBC) and lymphocyte counts—excluded in the recent update—could further improve its performance. The present study aimed to validate SOFA-2 in patients with sepsis and to examine its performance following integration of supplementary immune markers.

**Methods:**

This study is a secondary analysis of a multi-center randomized controlled trial in adult patients with sepsis conducted in China from September 2016 to December 2020. Both versions of SOFA were re-calculated at randomization, with an XGBoost-derived extension that incorporated additional immune markers, including WBC, lymphocyte counts, monocyte human leucocyte antigen-DR, neutrophil-to-lymphocyte ratio, and percentage of regulatory T cells. The discriminatory performance for predicting intensive care unit (ICU) mortality, evaluated by the area under the receiver operating characteristic curve (AUC), was examined.

**Results:**

Among the 1089 patients with sepsis (median age 64.5 years; 31.1% female), 9.2% died during ICU stay. The SOFA-2 was lower than the SOFA-1 (median [interquartile range] 6 [4–9] *vs*. 7 [5–10], *P*=0.001), primarily due to lower scores in the respiratory, cardiovascular, and liver domains, although higher values were observed in the kidney domain. Using a cut-off of 2, the two scores demonstrated highly concordant distributions; only 2.2% of patients had a SOFA-1 ≥2 but a SOFA-2 <2. The two scores demonstrated comparable discriminatory ability for predicting ICU mortality, with AUCs of 0.646 (95% confidence interval 0.595–0.698) for SOFA-2 and 0.641 (0.587–0.696) for SOFA-1. Incorporating any combination of the five immune markers into SOFA-2 did not result in significant changes in AUC.

**Conclusions:**

Updating from SOFA-1 to SOFA-2 results in comparable score distributions and sustains the discriminatory capacity for predicting ICU mortality in patients with sepsis. Immune dysregulation in sepsis may be too complex to capture with simple baseline markers.

**Trial registration:**

clinicaltrials.gov NCT02867267.

## Introduction

The Sequential Organ Failure Assessment (SOFA) score, initially developed in 1994 and published in 1996,^[^^1^^]^ has become a widely used scoring system in intensive care. Unlike other disease-severity indices that focus primarily on mortality prediction, the SOFA score was designed to assess and monitor organ dysfunction in critically ill patients. As a result, it plays a central role in the diagnosis and management of sepsis, which is defined as “life-threatening organ dysfunction caused by a dysregulated host response to infection.”^[^^2^^]^ Since intensive care practice has evolved substantially over the past 30 years,^[^^3^^]^ the score was recently updated to the SOFA-2 score to better reflect contemporary practice while maintaining its predictive performance.^[^^4^^]^ The main changes include the addition of updated organ-support drugs and devices, revised score cut-offs aligned with mortality risk, and inclusion of alternative variables for situations where primary measures are unavailable or not indicated.^[^^5^^]^

Although the SOFA-2 score has been evaluated in general (case-mix) adult critically ill patients—specifically, across ten cohorts with an intensive care unit (ICU) mortality ranging from 4.0% to 20.5%—it has not been specifically evaluated in patients with sepsis. Given that the SOFA-2 score may differ from the original SOFA (referred to as SOFA-1) score in magnitude (i.e., values of SOFA-2 compared to SOFA-1 are higher in 11% but lower in 40% of patients^[^^6^^]^), it is necessary to determine how the two scores differ when applying the diagnostic criteria for sepsis, where a key criterion is an acute increase of ≥2 SOFA points.^[^^2^^]^ Indeed, the developers of the updated SOFA-2 score explicitly noted that, in the context of sepsis diagnosis, whether SOFA-2 should replace SOFA-1 to better reflect current practice remains unresolved.^[^^5^^]^ In addition, although one of the major motivations for updating the SOFA score was to incorporate immune dysfunction (alongside gastrointestinal dysfunction), the immune domain was ultimately excluded due to insufficient content validity of the available markers (white blood cell [WBC] and lymphocyte counts).^[^^5^^]^

To help address these important knowledge gaps, we used data from the recently published TESTS trial^[^^7^^]^ to compare score distributions and predictive performance between the SOFA-2 and SOFA-1 scores, and to explore whether incorporating additional immune markers using a flexible modeling framework could further improve the performance of the SOFA-2 score.

## Methods

### Data source and study population

This study is a secondary analysis of data from the TESTS trial (NCT02867267), which was designed to evaluate the efficacy and safety of thymosin α1, an immunomodulatory agent, in adults with sepsis. The trial enrolled 1106 patients aged 18–85 years who were admitted to ICUs with a diagnosis of sepsis according to the sepsis-3 criteria.^[^^2^^]^ Participants were recruited from 22 centers in China between September 2016 and December 2020, and were randomly assigned in a 1:1 ratio to receive subcutaneous injection of thymosin α1 (*n*=552) or placebo (*n*=554) every 12 h for 7 days. Key exclusion criteria included pregnancy or lactation; hematological malignancy; prior organ or bone marrow transplantation; acute autoimmune disease or glomerulonephritis; thymosin α1 allergy; recent cardiopulmonary resuscitation with poor neurological recovery (Glasgow Coma Scale [GCS] ≤8); recent radiotherapy, chemotherapy, immunosuppressive therapy, or steroid use >10 mg/day; recent participation in immunity-related trials; undrained infectious foci; an underlying disease expected to be fatal within 28 days; or a family decision to forgo life-sustaining treatment. The original trial protocol was approved by the ethics committee of The First Affiliated Hospital of Sun Yat-sen University (No. 2016007), and details of the trial results have been published previously.^[^^7^^]^

For the present secondary analysis, we obtained only de-identified data from participants included in the modified intention-to-treat analysis (*n*=1089 in total; *n*=542 and 547 in the thymosin α1 and placebo arm, respectively).^[^^7^^]^ Compared with the 1106 randomized participants, 17 patients were excluded due to withdrawal of consent before receiving the allocated intervention.

### Baseline characteristics including immune markers

The day of randomization (Day 0) was considered the baseline for the current study. The following variables, closest to baseline when available, were included in the analysis: demographics, comorbidities, information about infection and microorganism, the Acute Physiology and Chronic Health Evaluation (APACHE) II, and certain laboratory results. The following variables are available and considered as immune markers: WBC and lymphocyte counts, monocyte human leucocyte antigen-DR (mHLADR), neutrophil-to-lymphocyte ratio (NLR), and percentage of regulatory T cells. More details on the full list of baseline characteristics, including their missingness, are provided in Supplementary e-Methods.

### Calculation of the SOFA scores

Both the SOFA-1 and SOFA-2 scores were calculated according to the methods described in the original literature.^[^^1^^,^^4^^]^ Comprehensive descriptions of all involved variables, including the complete data-processing and calculation workflow, are presented in Supplementary e-Methods.

### Clinical outcomes

ICU mortality was studied as the primary outcome in the present study, with 28-day mortality and 90-day mortality evaluated as secondary outcomes. Additional clinical outcomes available in the trial dataset (e-Methods), including length of stay in ICU or hospital, number of days free from mechanical ventilation, vasopressors, or continuous renal replacement, and quality of life, were summarized descriptively only.

### Statistical analysis

Baseline characteristics, including variables used in the calculation of SOFA scores, were summarized as median (interquartile range [IQR]) for continuous variables and frequency (percentage) for categorical variables. Distributions of the SOFA scores were visualized using heat maps and compared between SOFA-1 and SOFA-2 overall and across individual organ domains. Group differences were assessed using Mann-Whitney U tests for continuous variables, and Pearson’s Chi-square tests or Fisher’s exact tests, as appropriate, for categorical variables. Standardized mean differences (SMDs) were also calculated. Participants were further cross-classified by levels of SOFA-1 and SOFA-2, using 2 as the cut-off (≥2 *vs.* <2). Baseline characteristics and all available clinical outcomes were described across these groups. For ICU mortality, 28-day and 90-day mortality, distributions by combined SOFA-1 and SOFA-2 categories were visualized, and the area under the receiver operating characteristic curve (AUC) for SOFA-1 and SOFA-2 in predicting each outcome was estimated separately.

Regarding the exploration of SOFA-2′s extension with additional immune markers, we first evaluated the AUCs after incorporating the originally proposed immune domain^[^^4^^]^—derived from WBC and lymphocyte counts only—into the SOFA-2 score. To further assess whether adding any combination of five candidate immune markers (31 total combinations) improved the prediction of ICU mortality, we compared two models for each combination: (1) a baseline logistic regression model using the original SOFA-2 score alone, and (2) an augmented model using XGBoost that included the SOFA-2 score and the selected immune marker(s). XGBoost hyperparameters were tuned separately for each combination using five-fold cross-validation over a predefined grid of “max_depth” and “nrounds”, with all other parameters held constant. Internal validation was performed using 2000 bootstrap resamples. In each iteration, a bootstrap training set and an out-of-bag test set were generated. Values of the immune markers were standardized within the training set and applied to the test set to avoid information leakage. Both logistic and XGBoost models were refit in every bootstrap sample, and AUCs were estimated in the corresponding out-of-bag sets. For each immune marker combination, we computed the mean AUC and 95% bootstrap percentile confidence intervals (CIs) for both the baseline and augmented models, as well as the mean change in AUC. Missing predictor values were not imputed for the XGBoost models, as this tree-based algorithm natively accommodates missing data by learning optimal split directions for observations with missing predictors. In contrast, baseline logistic regression models were fitted using complete-case analysis.

All analyses were conducted in R (version 4.5.1). Statistical significance was defined as a two-sided α level of 0.05. The R code for the primary analyses of adding immune markers to SOFA-2 is included in the Supplementary Material (e-Methods).

## Results

### Patient characteristics

**Figure 1:**
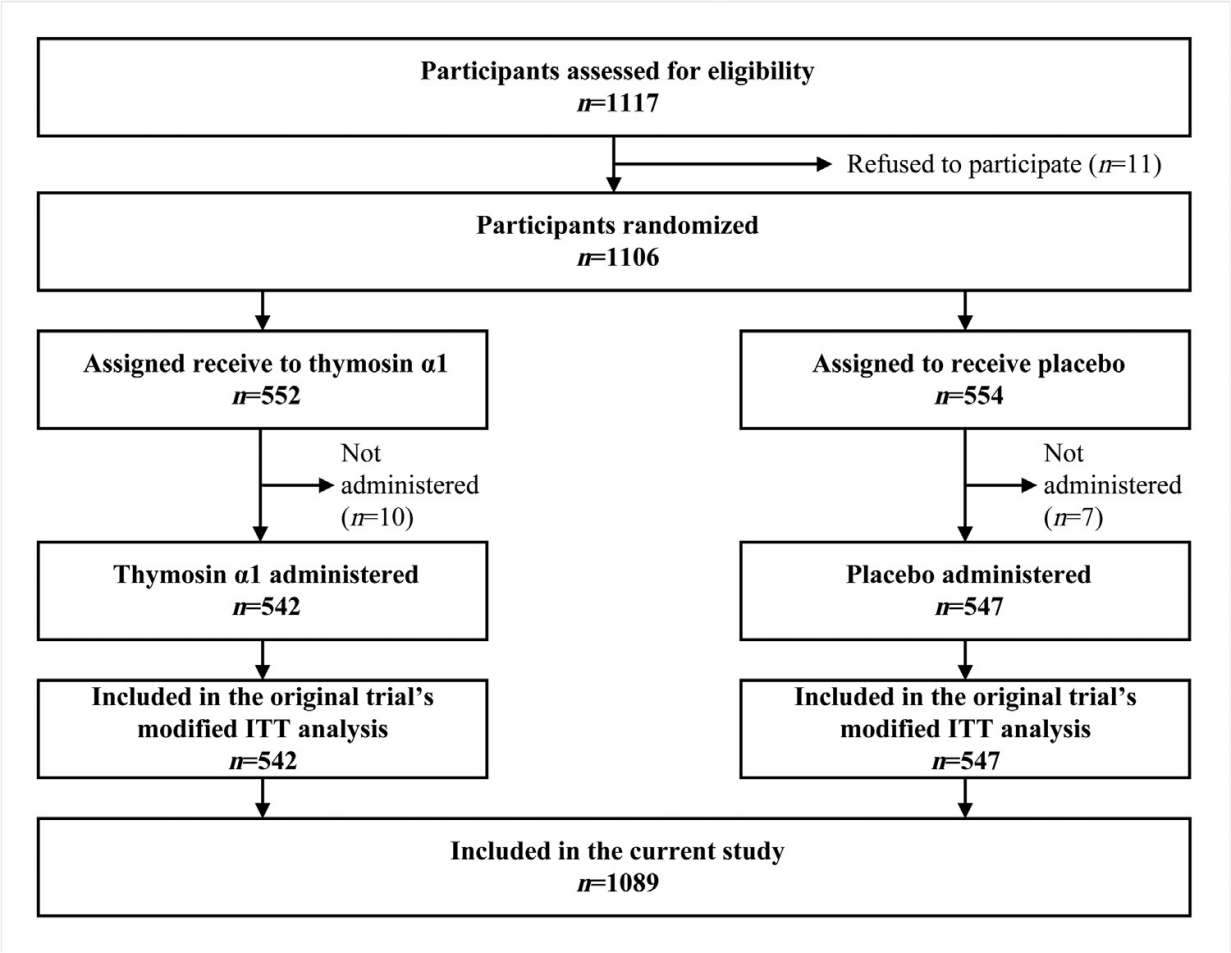
Flow diagram of the study participants. ITT: Intention-to-treat.

Among the 1089 patients with sepsis included in the study (Figure 1), the median age was 64.5 years (IQR: 51.5–73.1), and 31.1% were female. Approximately half (49.4%) were admitted to the ICU following a surgical procedure. Hypertension was the most common pre-existing condition (38.7%), followed by diabetes mellitus (25.4%). With respect to infection sites, aside from multiple (19.8%) or unknown (21.2%) locations, the most frequent were lung (31.5%) and abdomen (9.9%). Gram-negative organisms (34.8%) and mixed pathogens (26.9%) were the most commonly identified microorganisms, although 21.4% of cases were culture-negative. The median APACHE II score in the study population was 14 (IQR: 10-19). The ICU mortality, 28-day and 90-day mortality were 9.2%, 23.8%, and 31.7%, respectively. Additional patient characteristics are presented in Supplementary e-Table 1. Summary statistics for all variables contributing to score calculation are also provided in Supplementary e-Table 2.

### Score distributions

**Figure 2:**
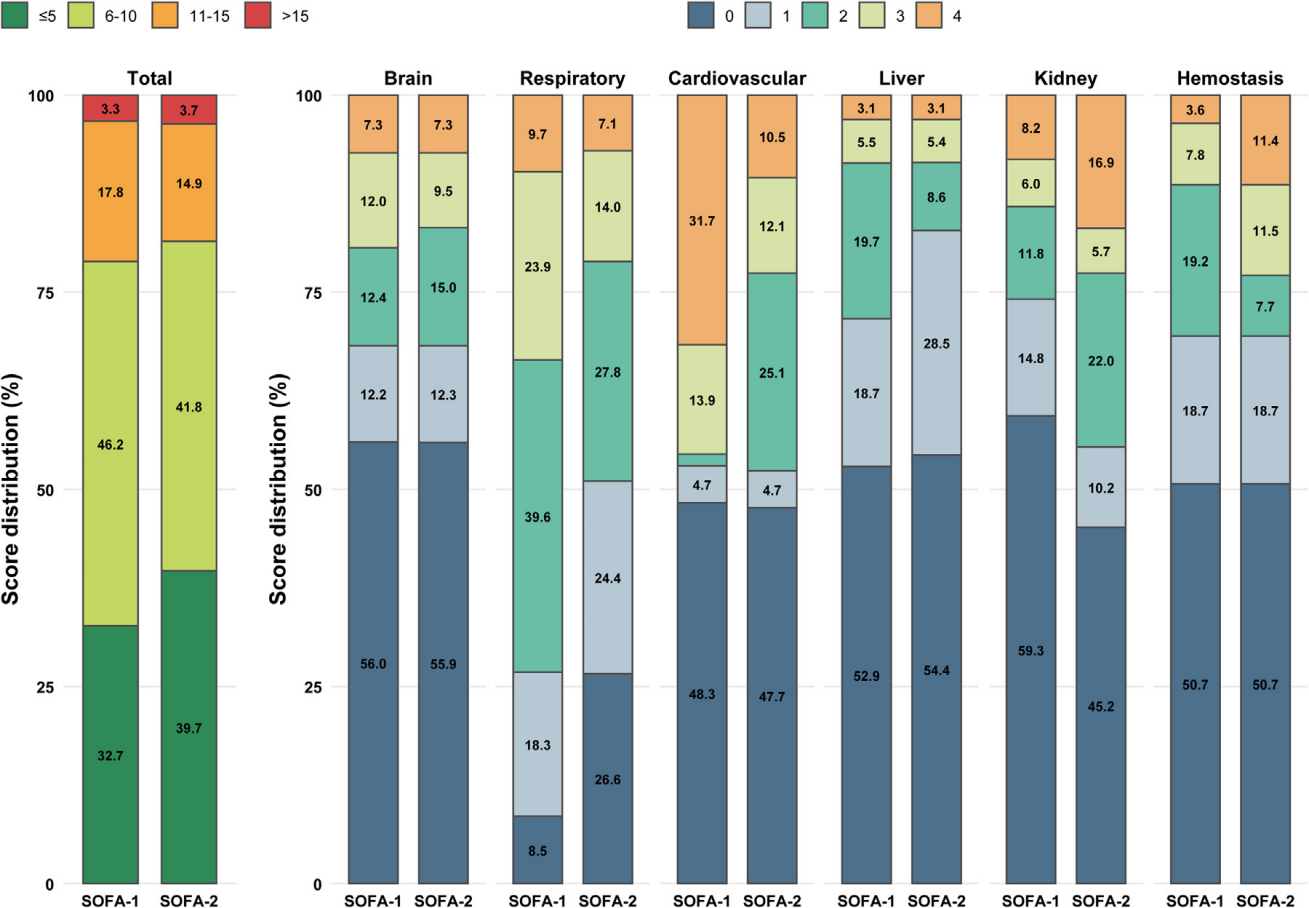
Distribution change from SOFA-1 to SOFA-2. The system names presented in the figure are from the SOFA-2 score. SOFA: Sequential organ failure assessment.

Compared with SOFA-1, SOFA-2 was statistically significantly lower overall (median [IQR], 6 [4–9] *vs*. 7 [5–10], *P*=0.001, SMD=0.120). As shown in Figure 2 and Supplementary e-Table 3, by organ domain, SOFA-2 was lower in the respiratory (1 [0–2] *vs*. 2 [1–3], *P* <0.001, SMD=0.501), cardiovascular (1 [0–2] *vs*. 1 [0–4], *P* <0.001, SMD=0.262), and liver (0 [0–1] *vs*. 0 [0–2], *P*=0.017, SMD=0.120) domains, but higher in the kidney domain (1 [0–2] *vs*. 0 [0–2], *P* <0.001, SMD=0.356). Although the hemostasis domain scores did not differ statistically between the two versions, a greater proportion of patients had a score above 2 in SOFA-2 than in SOFA-1 (Supplementary e-Table 3).

**Figure 3:**
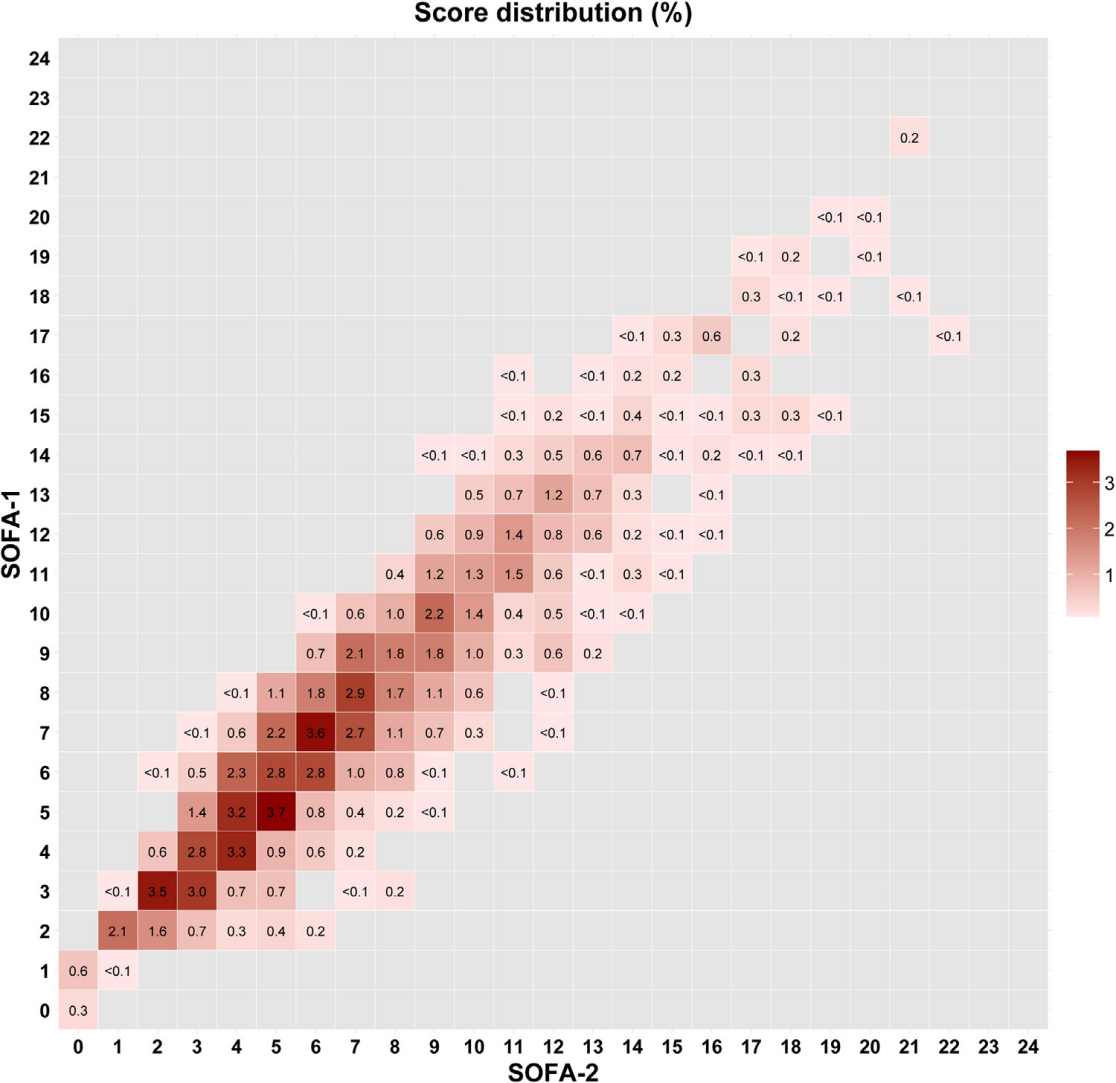
Reclassification between SOFA-1 and SOFA-2. The percentage inside each cell represents the proportion of patients in that cell compared with the total. Gray cells denote no data. SOFA: Sequential organ failure assessment.

The distribution of SOFA-2 by SOFA-1 is shown in Figure 3, demonstrating highly concordant distributions. When patients were cross-classified using a binary threshold (≥2 *vs*. <2) for both scores, 96.8% had both scores ≥2, 1.0% had both scores <2, and 2.2% had a SOFA-1 ≥2 but a SOFA-2 <2. No patients had a SOFA-1 <2 with a SOFA-2 ≥2. Baseline characteristics and clinical outcomes of these groups are summarized in Supplementary e-Tables 4 and 5.

### Discriminatory power

**Figure 4:**
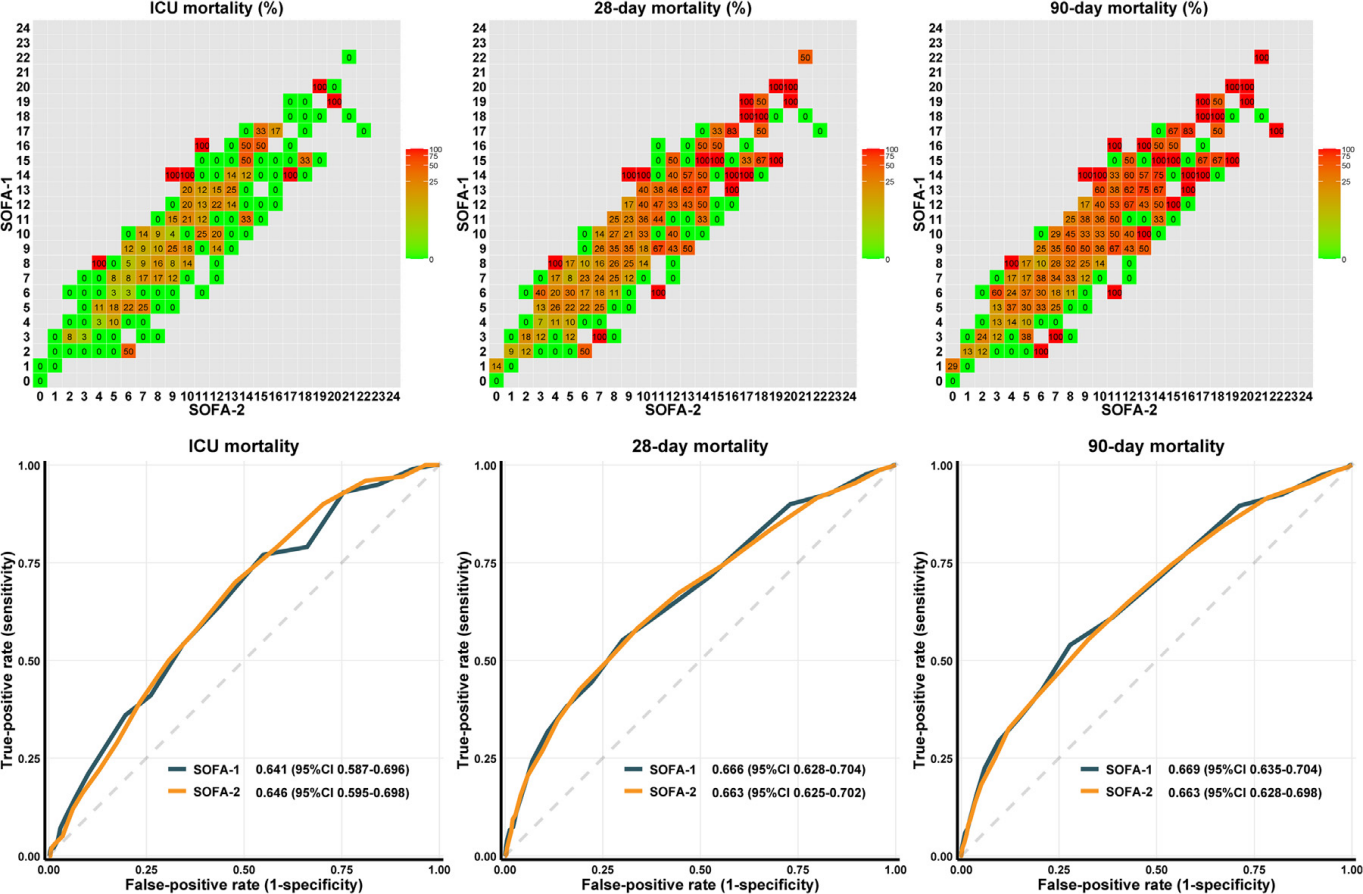
Discriminatory power for mortality between SOFA-1 and SOFA-2. The percentage inside each cell represents mortality. Gray cells denote no data. SOFA: Sequential organ failure assessment; ICU: Intensive care unit; CI: Confidence interval.

As shown in Figure 4, higher SOFA scores were associated with a trend toward increased ICU mortality. The two versions of SOFA scores demonstrated comparable discriminatory performance (SOFA-2: AUC=0.646, 95%CI: 0.595 to 0.698; *vs*. SOFA-1: AUC=0.641, 95%CI: 0.587 to 0.696). Similar patterns were observed for 28-day mortality (AUC [95%CI], 0.663 [0.625–0.702] *vs*. 0.666 [0.628–0.704]) and 90-day mortality (0.663 [0.628–0.698] *vs*. 0.669 [0.635–0.704]).

### Adding immune markers to SOFA-2

Incorporating the originally proposed immune domain (i.e., WBC and lymphocyte counts) into SOFA-2 did not result in significant changes in AUCs for predicting ICU mortality, 28-day mortality, or 90-day mortality (Supplementary e-Figure 1). When three additional immune markers (i.e., mHLADR, NLR, and percentage of regulatory T cells) were included as candidate predictors, integrating any combination of these five markers using an XGBoost algorithm likewise did not significantly change the AUC for predicting ICU mortality (Table 1).

**Table 1:** Predictive performance of SOFA-2 score augmented with different immune markers for ICU mortality.

Additional predictors (immune markers) added to SOFA-2	Max tree depth	Iteration (nrounds)	AUC of SOFA-2 for predicting ICU mortality (95%CI)	AUC of SOFA-2 plus the additional predictors for predicting ICU mortality (95%CI)	Difference in AUC (95%CI)
WBC	2	31	0.645 (0.572 to 0.712)	0.622 (0.537 to 0.698)	−0.024 (−0.116 to 0.038)
Lym	3	50	0.646 (0.577 to 0.715)	0.608 (0.507 to 0.682)	−0.039 (−0.153 to 0.040)
mHLADR	3	11	0.647 (0.578 to 0.716)	0.593 (0.515 to 0.666)	−0.055 (−0.133 to 0.001)
NLR	2	50	0.647 (0.578 to 0.713)	0.604 (0.526 to 0.673)	−0.043 (−0.127 to 0.022)
Treg	2	25	0.647 (0.577 to 0.716)	0.602 (0.508 to 0.679)	−0.045 (−0.136 to 0.018)
WBC + Lym	4	25	0.646 (0.578 to 0.710)	0.623 (0.540 to 0.697)	−0.023 (−0.113 to 0.048)
WBC + mHLADR	4	122	0.645 (0.581 to 0.713)	0.614 (0.542 to 0.684)	−0.031 (−0.120 to 0.047)
WBC + NLR	4	79	0.647 (0.579 to 0.714)	0.629 (0.552 to 0.699)	−0.018 (−0.105 to 0.057)
WBC + Treg	5	17	0.646 (0.578 to 0.716)	0.611 (0.512 to 0.697)	−0.035 (−0.135 to 0.046)
Lym + mHLADR	4	60	0.646 (0.579 to 0.716)	0.595 (0.499 to 0.667)	−0.051 (−0.147 to 0.019)
Lym + NLR	2	151	0.646 (0.577 to 0.711)	0.613 (0.513 to 0.685)	−0.032 (−0.130 to 0.039)
Lym + Treg	3	18	0.646 (0.580 to 0.714)	0.608 (0.517 to 0.686)	−0.039 (−0.135 to 0.036)
mHLADR + NLR	2	23	0.646 (0.576 to 0.717)	0.596 (0.514 to 0.670)	−0.051 (−0.136 to 0.012)
mHLADR + Treg	2	13	0.645 (0.570 to 0.712)	0.597 (0.497 to 0.682)	−0.049 (−0.155 to 0.023)
NLR + Treg	4	49	0.646 (0.577 to 0.712)	0.608 (0.509 to 0.688)	−0.038 (−0.140 to 0.041)
WBC + Lym + mHLADR	3	94	0.646 (0.576 to 0.714)	0.632 (0.555 to 0.704)	−0.015 (−0.101 to 0.061)
WBC + Lym + NLR	5	66	0.647 (0.577 to 0.718)	0.625 (0.549 to 0.697)	−0.022 (−0.114 to 0.058)
WBC + Lym + Treg	3	185	0.646 (0.576 to 0.712)	0.629 (0.556 to 0.701)	−0.017 (−0.111 to 0.063)
WBC + mHLADR + NLR	4	93	0.647 (0.575 to 0.714)	0.619 (0.548 to 0.691)	−0.028 (−0.114 to 0.050)
WBC + mHLADR + Treg	2	92	0.647 (0.576 to 0.719)	0.624 (0.550 to 0.700)	−0.023 (−0.108 to 0.047)
WBC + NLR + Treg	4	88	0.647 (0.578 to 0.715)	0.632 (0.555 to 0.706)	−0.015 (−0.104 to 0.061)
Lym + mHLADR + NLR	2	167	0.647 (0.579 to 0.716)	0.607 (0.525 to 0.677)	−0.040 (−0.140 to 0.035)
Lym + mHLADR + Treg	2	45	0.646 (0.578 to 0.715)	0.609 (0.515 to 0.682)	−0.036 (−0.129 to 0.029)
Lym + NLR + Treg	4	81	0.646 (0.580 to 0.712)	0.611 (0.497 to 0.689)	−0.035 (−0.140 to 0.046)
mHLADR + NLR + Treg	2	96	0.647 (0.577 to 0.714)	0.602 (0.512 to 0.679)	−0.045 (−0.138 to 0.024)
WBC + Lym + mHLADR + NLR	2	117	0.647 (0.576 to 0.716)	0.631 (0.554 to 0.703)	−0.016 (−0.103 to 0.058)
WBC + Lym + mHLADR + Treg	2	151	0.645 (0.578 to 0.712)	0.636 (0.560 to 0.709)	−0.010 (−0.096 to 0.065)
WBC + Lym + NLR + Treg	5	82	0.647 (0.580 to 0.712)	0.630 (0.554 to 0.705)	−0.016 (−0.109 to 0.060)
WBC + mHLADR + NLR + Treg	2	91	0.647 (0.581 to 0.715)	0.624 (0.549 to 0.695)	−0.024 (−0.116 to 0.049)
Lym + mHLADR + NLR + Treg	4	98	0.646 (0.574 to 0.714)	0.598 (0.491 to 0.672)	−0.048 (−0.150 to 0.031)
WBC + Lym + mHLADR + NLR + Treg	5	96	0.647 (0.578 to 0.716)	0.622 (0.546 to 0.696)	−0.025 (−0.119 to 0.056)

AUC for the SOFA-2 score alone was derived from a logistic regression model. Models including SOFA-2 score plus immune predictors were fitted using XGBoost. The following parameters were held fixed: binary logistic objective, AUC as the evaluation metric, learning rate 0.05, subsample 0.8, colsample_bytree 0.8, and min_child_weight 1. Hyperparameter optimization was limited to maximum tree depth and the optimal number of boosting rounds (nrounds), selected via five-fold cross-validation. Model performance estimates (AUC and AUC differences) were obtained from 2000 bootstrap resamples.

SOFA: Sequential organ failure assessment; ICU: Intensive care unit; AUC: Area under the receiver operating characteristic curve; CI: Confidence interval; WBC: White blood cell; Lym: Lymphocyte count; mHLADR: Monocyte human leucocyte antigen-DR; NLR: Neutrophil-to-lymphocyte ratio; Treg: Regulatory T cells.

## Discussion

In this secondary analysis of a clinical trial in sepsis, we found that the updated SOFA-2 score maintained its score distribution and preserved its discriminatory ability for predicting ICU mortality in patients with sepsis. Neither the originally proposed immune domain nor a machine-learning-based integration of multiple available immune markers yielded additional improvement. This first-time validation suggests that SOFA-2 may supersede SOFA-1 in the context of sepsis, providing better alignment with current clinical practice. Furthermore, our seemingly unsuccessful attempt to enhance the score with supplementary immune markers highlights the complexity of immune dysregulation and underscores the need for further investigation.

To our knowledge, no prior study has specifically evaluated the SOFA-2 score in sepsis. Although such an analysis could, in principle, be conducted retrospectively using electronic health record data, identifying sepsis cases retrospectively is challenging due to issues such as undercoding and diagnostic delays,^[^^8^^,^^9^^]^ which may limit the interpretability of any resulting findings. A key strength of our evaluation study is the use of recent trial data, in which all sepsis cases were rigorously adjudicated and closely aligned with contemporary diagnostic and management practices. Furthermore, because all variables (including those required for score calculation) were prospectively collected, the SOFA scores in our study may be less susceptible to measurement error. Some compromises were unavoidable, however, due to unavailable variables or insufficient data granularity (see Supplementary e-Methods). Consistent with the study that developed SOFA-2,^[^^4^^]^ we observed domain-level and total score shifts in the same directions when transitioning from SOFA-1 to SOFA-2, although the SOFA scores in our study cohort were higher in absolute terms. These consistent patterns of score shifts are expected, given that a major component of the update from SOFA-1 to SOFA-2 involved revising score cut-offs.

A noteworthy methodological point concerns the metric used to evaluate the performance of the SOFA scores. In both the original study that developed the SOFA-2^[^^4^^]^ and in our analysis, discrimination for predicting ICU mortality, assessed using the AUC, was chosen to compare performance between the two score versions. Conceptually, however, this represents a mismatch between what the metric measures and what the score is intended to quantify. The SOFA score was designed to capture the severity of organ dysfunction, yet its development relied on the association between physiologic variables and mortality. Although mortality is closely linked to the degree of organ dysfunction—and therefore should correlate with any accurate measure of that dysfunction—the score inevitably incorporates elements of mortality risk rather than reflecting pure pathophysiologic dysfunction. This may limit its pathophysiologic specificity, for example, in cases of isolated organ failure.^[^^10^^]^ Nonetheless, because the SOFA scores are widely embedded in daily ICU practice, the lack of a significant change in AUC when moving from SOFA-1 to SOFA-2 should not be interpreted as evidence that the update was unnecessary. Rather, the revision aims to ensure that the score better reflects contemporary clinical practice.^[^^4^^]^

One contribution of our study is the demonstration that the updated SOFA-2 score shows a distribution highly consistent with the original SOFA-1 score when applying the binary cut-off of 2. This confirmation is clinically relevant in the context of sepsis, because a change in SOFA-1 score ≥2 is a key diagnostic criterion for sepsis,^[^^2^^]^ and it is common to assume a baseline SOFA score of 0. Excluding the 1% of patients whose SOFA-1 and SOFA-2 scores were both below 2—who likely experienced a decline in SOFA at the time of randomization—most patients consistently met the ≥2 threshold in both scoring versions. These findings support the use of the updated SOFA-2 in patients with sepsis. Patients with sepsis who showed discordant SOFA levels between SOFA-1 and SOFA-2 warrant further investigation, as they may represent a subgroup that might require a revised diagnostic cut-off. Because this subgroup was small in our cohort (*n*=24), we limited our evaluation to descriptive analyses. Notably, additional studies are also needed to evaluate cases in which SOFA-1 is low while SOFA-2 is high. This subgroup was excluded from our study population by design and therefore could not be examined here.

Another contribution of our study is the exploration of incorporating immune markers into the SOFA-2 score. As highlighted in the definition of sepsis,^[^^2^^]^ immune dysregulation has long been recognized as central to the pathobiology of sepsis.^[^^11^^,^^12^^]^ Although adding an immune domain was initially proposed during the update of the SOFA-2 score,^[^^4^^]^ it was ultimately omitted due to concerns regarding content validity. Our analysis provides insight into which immune markers could be incorporated and how best to do so. The immune domain proposed in the original development of SOFA-2 included only WBC and lymphocyte counts. We expanded this by integrating three additional immune markers using an advanced modeling approach. Although the associations of WBC^[^^13^^,^^14^^]^ and lymphocyte^[^^15^^,^^16^^]^ counts with prognosis in critically ill patients have been extensively studied, these markers were deemed to lack sufficient content validity: WBC may reflect a normal response to inflammatory insult, and immune dysfunction can occur despite normal leukocyte counts.^[^^5^^]^ Among the three additional markers we incorporated, mHLADR directly reflects monocyte deactivation, a hallmark of immune suppression in sepsis,^[^^17^^]^ and has been associated with patient outcomes in multiple studies.^[^[18], [19], [20]^]^ Numerous investigations have also supported the prognostic value of NLR,^[^^21^^]^ which correlates with presepsin,^[^^22^^]^ an innate immune response marker in sepsis.^[^^23^^]^ Regulatory T cells, likewise, are directly involved in immunosuppression after the onset of sepsis.^[^^24^^,^^25^^]^ Because the relationships between these markers and mortality are likely nonlinear—an issue that traditional statistical models handle poorly, particularly in the presence of missing data—we employed the XGBoost algorithm to integrate these new variables. The absence of a substantial improvement in the discriminatory performance of SOFA-2 after adding these immune markers may reflect the inherently dynamic and multidimensional nature of immune dysfunction, which cannot be fully captured by a limited set of markers or condensed into a single score. Alternatively, discriminatory power for mortality may not be the optimal metric to evaluate the value of incorporating these markers, given that many immune indicators exhibit only weak or inconsistent correlations with mortality, unlike organ dysfunction which itself is strongly linked to patient prognosis. Future work is needed to develop practical, multimodal tools for comprehensive immune assessment.^[^^11^^]^

## *Limitations*

The following limitations should be considered when interpreting our findings. First, although our analysis is based on clinical trial data, which are generally of high quality, some degree of missing data and measurement error is unavoidable, albeit likely limited. Several variables required to calculate the SOFA-2 score were either unavailable or lacked sufficient granularity (e.g., urine output). In addition, the SOFA scores were retrospectively reconstructed using variables measured closest to randomization rather than at ICU admission. These limitations may partly explain why the discriminatory performance of the SOFA scores in our study (AUC ≈ 0.65) was lower than that reported in prior studies (AUC above 0.7),^[^^2^^,^^4^^,^^26^^]^ although at least one previous report described an AUC comparable to ours.^[^^27^^]^ Therefore, additional studies are warranted to confirm our findings. Second, we evaluated the SOFA scores at baseline only, rather than as time-dependent measures. Dynamic assessment may provide important insights^[^^28^^]^ and should be investigated in future research. Third, potential selection bias may arise from the trial’s exclusion criteria, some of which may have restricted scores of certain organ domains to a narrower range. Fourth, as discussed above, determining the value of incorporating an immune marker solely from its effect on the AUC for predicting ICU mortality may be debatable. Fifth, our modeling strategy did not include an external validation cohort, and therefore the findings should be interpreted as hypothesis-generating, but not evidence that the immune markers lack clinical relevance in the context of sepsis. Finally, our study cohort was limited to patients who met the sepsis-3 criteria. Consequently, findings related to the performance of the SOFA-2 score and the absence of incremental improvement from immune biomarker augmentation may not be generalizable to broader ICU patient populations. Therefore, our results should be interpreted cautiously when applied to populations beyond sepsis-3-defined sepsis, such as patients with severe infection lacking sepsis-3-defined organ dysfunction or those with non-infectious critical illness.

## Conclusions

Updating from SOFA-1 to SOFA-2 results in comparable score distributions and sustains the discriminatory capacity for predicting ICU mortality in patients with sepsis. Immune dysregulation in sepsis may be too complex to capture with simple baseline markers.

## Acknowledgment

The authors thank all study participants and investigators of the TESTS trial.

## Funding

This study was supported by the 10.13039/501100001809National Natural Science Foundation of China (No. 82302415 to FP, No. 82002076 to BG, No. 82272186 to XG, No. 82472221 to JW), Guangdong Provincial Enterprise Joint Foundation for Basic and Applied Basic Research Key program Project (No. 2024B1515230008 to XG, No. 2023B1515230005 to JW), and the Guangdong Clinical Research Center for Critical Care Medicine (No. 2020B1111170005). QC is supported by the “100 Top Talents Program” from Sun Yat-sen University.

## Ethics Statement

The original trial was approved by the ethics committee of The First Affiliated Hospital of Sun Yat-sen University (No. 2016007) in China.

## Conflict of Interest

JW is an Editorial Board Member for this journal and was not involved in the editorial review or the decision to publish this article. All authors declare that they have no known competing financial interests or personal relationships that could have appeared to influence the work reported in this paper.

## Data Availability

The datasets used and/or analyzed during the current study are available from the corresponding authors on reasonable request.

## CRediT authorship contribution statement

**Fei Pei:** Writing – review & editing, Validation, Investigation, Formal analysis. **Bin Gu:** Writing – review & editing, Validation, Investigation, Formal analysis. **Zimeng Liu:** Writing – review & editing, Validation, Investigation, Formal analysis. **Guangzhen Li:** Writing – review & editing, Project administration, Data curation. **Yao Nie:** Writing – review & editing. **Minying Chen:** Writing – review & editing. **Yongjun Liu:** Writing – review & editing. **Xiangdong Guan:** Writing – review & editing. **Qingui Chen:** Writing – original draft, Methodology, Formal analysis, Data curation. **Jianfeng Wu:** Writing – review & editing, Validation, Supervision, Resources, Funding acquisition, Conceptualization.

## Appendix Supplementary materials

Image, application 1
