# Supplementary material for: Validation of SOFA-2 score in sepsis and exploration of its extension with additional immune markers
Source: J Intensive Med. 2026 Jan 8;6(3):294–301. doi: 10.1016/j.jointm.2025.12.003 (PMC13184492; doi:10.1016/j.jointm.2025.12.003)
Supplement: Supplementary file 1 [file mmc1.pdf]

Supplemental material

e-Methods	1. Variables used in the study	2
e-Methods	2. R code for the primary analyses of adding immune markers to SOFA-2	11
e-Table 1	Baseline characteristics of the study participants	16
e-Table 2	Summary statistics about baseline characteristics involved in SOFA score calculation	18
e-Table 3	Comparison of SOFA-1 and SOFA-2 scores	19
e-Table 4	Baseline characteristics of the study participants by level of SOFA score	21
e-Table 5	Clinical outcomes of the study participants by level of SOFA score	23
e-Figure 1	Discriminatory power for mortality between SOFA-2 and SOFA-2 augmented with the immune domain	24

e-Methods

1. Variables Used in the Study

Raw data were obtained for all participants in the TESTS trial (NCT02867267; PMID 39814420) who were included in the modified intention-to-treat analysis (n=1089), including baseline characteristics, variables relevant to SOFA score calculation, and clinical outcomes.

**Variable**	**Details**
**Baseline characteristics**	
Age	Unit: years; No missing value
Age group	<60 vs. ≥60 years, newly created by the variable age; No missing value
Sex	Male vs. Female; No missing value
Height	Unit: centimeter; 472 (43.3%) values are missing.
Weight	Unit: kilogram; 453 (41.6%) values are missing. The variable weight was imputed as part of estimating urine output in units of mL/kg/h. Further details are provided below.
Admission to ICU after a surgical procedure	Yes vs. No; No missing value
Hypertension (pre-existing conditions)	Yes vs. No; No missing value
Coronary heart disease (pre-existing conditions)	Yes vs. No; No missing value
Chronic obstructive pulmonary disease (pre-existing conditions)	Yes vs. No; No missing value
Diabetes mellitus (pre-existing conditions)	Yes vs. No; No missing value
Chronic kidney disease (pre-existing conditions)	Yes vs. No; No missing value
Solid malignant tumors (pre-existing conditions)	Yes vs. No; No missing value
Infection sites	Categories: Lung; Abdomen; Bloodstream; Urinary tract; Biliary tract; Skin and soft tissue; Others; Multiple sites; Unknown; 4 (0.4%) values were missing and recoded as a new category “Missing”.
Microorganisms	Categories: Gram negative; Gram positive; Fungi; Atypical pathogens; Mixed; Culture negative; 4 (0.4%) values were missing and recoded as a new category “Missing”.
APACHE II score	No missing value
White blood cell (other laboratory results)	Unit: $\times 10^9$/L; 2 (0.2%) values are missing.
Lymphocyte count (immune marker)	Unit: $\times 10^9$/L; Normal reference >1.1$\times 10^9$/L; No missing value
Monocyte human leucocyte antigen-DR (immune marker)	Unit: antibodies per cell; Normal reference >15,000 antibodies per cell; 230 (21.1%) values are missing.
Neutrophil-to-lymphocyte ratio (immune marker)	Normal reference range 1-3; 1 (0.1%) value is missing.
Regulatory T cells (immune marker)	Unit: %; Normal reference <7.7%; 228 (20.9%) values are missing.
Neutrophil count (other laboratory results)	Unit: $\times 10^9$/L; 1 (0.1%) value is missing.
C reactive protein (other laboratory results)	Unit: mg/L; 257 (23.6%) values are missing.
Procalcitonin (other laboratory results)	Unit: ng/mL; 180 (16.5%) values are missing.
Lactate (other laboratory results)	Unit: mmol/L; 601 (55.2%) values are missing.
Prothrombin time (other laboratory results)	Unit: second; 18 (1.7%) values are missing.
Activated partial thromboplastin time (other laboratory results)	Unit: second; 18 (1.7%) values are missing.
International normalized ratio (other laboratory results)	19 (1.7%) values are missing.
D-dimer (other laboratory results)	Unit: ng/mL FEU; 120 (11.0%) values are missing.

Treatment arm assigned in the original trial	Placebo vs. Thymosin alpha1; No missing value
**Variables relevant to SOFA score calculation**	
Central nervous system (original SOFA-1 from trial dataset)	1 (0.1%) value is missing.
Glasgow Coma Scale	No missing value, but 1 (0.1%) record contained the value "1+2+T", which was treated as missing. Motor-scale domain is unavailable.
Drugs to treat delirium<br>(Yes vs. No)	Information on the use of other medications (i.e., in addition to Norepinephrine, Epinephrine, Dopamine, Dobutamine, and the study intervention; recorded as free text) is screened for the following medications: Dexmedetomidine, Haloperidol, Olanzapine, Quetiapine, and Ziprasidone.<br><br>For treatments whose start or end dates include the baseline (i.e., Day 0), additional criteria were applied. For Dexmedetomidine to be classified as treatment for delirium, the route of administration must be intravenous (IV) with continuous infusion, and the recorded indication must be related to delirium rather than sedation. For Haloperidol, Olanzapine, Quetiapine, and Ziprasidone, the recorded indication must be related to delirium (but not Schizophrenia or bipolar disorder).<br><br>After applying these criteria, only 1 record of Olanzapine use was classified as treatment for delirium.
Central nervous system (SOFA-1)	0: Glasgow Coma Scale =15 or missing<br>1: Glasgow Coma Scale 13-14<br>2: Glasgow Coma Scale 10-12<br>3: Glasgow Coma Scale 6-9<br>4: Glasgow Coma Scale <6
Central nervous system (SOFA-2)	0: Glasgow Coma Scale =15 or missing<br>1: Glasgow Coma Scale 13-14<br>2: Glasgow Coma Scale 9-12<br>3: Glasgow Coma Scale 6-8<br>4: Glasgow Coma Scale <6<br><br>After applying the above condition to calculate the score, if a record includes a drug used to treat delirium (only Olanzapine appears in the data) but its score is 0, then update the score to 1.
Respiration (original SOFA-1 from trial dataset)	8 (0.7%) values are missing
PaO_2	4 values were missing. The data were originally reported in a mixture of kPa (n=73) and mmHg. To standardize units, values in kPa were converted to mmHg by multiplying by 7.5. After conversion, any PaO_2 <45 or >500 mmHg was classified as an extreme value and treated as missing (n=32). Following this data processing, a total of 36 (3.3%) values are missing.
FiO_2	6 values were missing. One record exceeded 1, which was assumed to have been entered as a percentage and was corrected by dividing by 100. Additionally, 2 records were <0.21, classified as extreme values, and treated as missing. After this data processing, 8 (0.7%) values are missing in total.

PaO_2: FiO_2 ratio	Unit: mmHg; 8 (0.7%) values are missing; Compared with the manually calculated PaO_2: FiO_2 ratio, there were 72 records in which both PaO_2 and FiO_2 were available but the absolute difference in the ratio exceeded 1. In these cases, the reported FiO_2: FiO_2 ratio was assumed to be valid and is used for subsequent score calculations.
Mechanical ventilation	Yes vs. No; No missing value<br>The original dataset does not contain a variable that differentiates noninvasive respiratory support (including specific subtypes) from invasive support; however, long-term home ventilation is likely excluded.
Respiration (SOFA-1)	0: PaO_2: FiO_2 ratio >400 mmHg, or PaO_2: FiO_2 ratio is missing<br>1: PaO_2: FiO_2 ratio >300 AND ≤400 mmHg<br>2: [PaO_2: FiO_2 ratio >200 AND ≤300 mmHg], or [PaO_2: FiO_2 ratio ≤200 AND Mechanical ventilation is "No"]<br>3: PaO_2: FiO_2 ratio ≤200 AND >100 mmHg, AND Mechanical ventilation is "Yes"<br>4: PaO_2: FiO_2 ratio ≤100 mmHg AND Mechanical ventilation is "Yes"
Respiratory (SOFA-2)	0: PaO_2: FiO_2 ratio >300 mmHg<br><br>1: PaO_2: FiO_2 ratio ≤300 AND >225 mmHg<br><br>2: PaO_2: FiO_2 ratio ≤225 AND >150 mmHg<br><br>3: PaO_2: FiO_2 ratio ≤150 AND >75 mmHg, AND Mechanical ventilation is "Yes"<br><br>4: PaO_2: FiO_2 ratio ≤75 AND Mechanical ventilation is "Yes"<br><br>2: PaO_2: FiO_2 ratio ≤150 mmHg, AND Mechanical ventilation is "No"<br><br>0: PaO_2: FiO_2 ratio is missing, AND Mechanical ventilation is "No"<br><br>3: PaO_2: FiO_2 ratio is missing AND Mechanical ventilation is "Yes"<br><br>Note that both arterial oxygen saturation (SpO_2) and extracorporeal membrane oxygenation are unavailable in the dataset. We assume that no advanced respiratory support was withheld due to a ceiling of treatment.
Cardiovascular (original SOFA-1 from trial dataset)	28 (2.6%) values are missing
Mean arterial pressure	Unit: mmHg; 22 (2.0%) values are missing; All values fell within the range 15-199 mmHg; therefore, no additional records were treated as missing. For calculations of Cardiovascular (SOFA-1) and Cardiovascular (SOFA-2), missing values were imputed as 90 mmHg.

Norepinephrine dose	Unit: μg/kg/min (expressed as the base); 36 (3.3%) values are missing, excluding an additional 567 records with missing values that, based on an accompanying dichotomous variable indicating the patient did not receive Norepinephrine, were imputed as a dose of 0. A small number of records contained values of "≥0.1" (n=1), ">0.1" (n=8), "≤0.1" (n=5), or "<0.1" (n=5), which were recoded to 0.2, 0.2, 0.05, and 0.05, respectively. For the calculation of Cardiovascular (SOFA-1) and Cardiovascular (SOFA-2), remaining missing values were imputed as 0.
Epinephrine dose	Unit: μg/kg/min; 6 (0.6%) values are missing, excluding an additional 1067 records with missing values that, based on an accompanying dichotomous variable indicating the patient did not receive Epinephrine, were imputed as a dose of 0. A small number of records contained values of "≥0.1" (n=1) or "≤0.1" (n=2), which were recoded to 0.2 and 0.05, respectively. For the calculation of Cardiovascular (SOFA-1) and Cardiovascular (SOFA-2), remaining missing values were imputed as 0.
Dopamine dose	Unit: μg/kg/min; 6 (0.6%) values are missing, excluding an additional 1055 records with missing values that, based on an accompanying dichotomous variable indicating the patient did not receive Dopamine, were imputed as a dose of 0. A small number of records contained values of "≤0.1" (n=3) or "≤5" (n=2), which were recoded to 0.05 and 2.5, respectively. For the calculation of Cardiovascular (SOFA-1) and Cardiovascular (SOFA-2), remaining missing values were imputed as 0.
Dobutamine dose	Unit: μg/kg/min; 7 (0.6%) values are missing, excluding an additional 1052 records with missing values that, based on an accompanying dichotomous variable indicating the patient did not receive Dobutamine, were imputed as a dose of 0. A small number of records contained values of "≤5" (n=2), which were recoded to 2.5. For the calculation of Cardiovascular (SOFA-1) and Cardiovascular (SOFA-2), remaining missing values were imputed as 0.
Other vasopressors or inotropes (Yes vs. No)	Information on the use of other medications (i.e., in addition to Norepinephrine, Epinephrine, Dopamine, Dobutamine, and the study intervention; recorded as free text) is screened for the following medications: Phenylephrine, Vasopressin, Metaraminol, Terlipressin, Midodrine, Milrinone, and Levosimendan.<br><br>Apart from treatments whose start or end dates include the baseline (i.e., Day 0), the route of administration must be intravenous (IV) with continuous infusion. After applying these criteria, only records of Terlipressin, Milrinone, and Levosimendan use were identified.<br><br>Other vasopressors or inotropes include Dopamine (dose >0), Dobutamine (dose >0), Terlipressin, Milrinone, and Levosimendan.
Cardiovascular (SOFA-1)	0: Mean arterial pressure ≥70 mmHg AND Dopamine dose =0 AND Dobutamine dose =0 AND Epinephrine dose =0 AND Norepinephrine dose =0<br><br>1: Mean arterial pressure <70 mmHg AND Dopamine dose =0 AND Dobutamine dose =0 AND Epinephrine dose =0 AND Norepinephrine dose =0

	4: Dopamine dose >15 µg/kg/min OR Epinephrine dose >0.1 µg/kg/min OR Norepinephrine dose >0.1 µg/kg/min<br><br>3: Dopamine dose >5 µg/kg/min OR [Epinephrine dose >0 AND Epinephrine dose ≤0.1 µg/kg/min] OR [Norepinephrine dose >0 AND Norepinephrine dose ≤0.1 µg/kg/min]<br><br>2: [Dopamine dose >0 AND Dopamine dose ≤5 µg/kg/min] OR Dobutamine dose >0<br><br>Note that when the medications were recorded with a dose greater than zero, they were assumed to have been administered for at least one hour, given the absence of hour-level administration data.
Cardiovascular (SOFA-2)	To help calculate the score, several variables were created:<br>(1) <u>Sum of Norepinephrine and Epinephrine dose</u>: Norepinephrine dose (µg/kg/min) + Epinephrine dose (µg/kg/min)<br><br>(2) <u>Other vasopressor or inotrope use</u>: Yes vs. No, where Yes = {Dopamine dose >0 OR Dobutamine dose >0 OR Terlipressin is "Yes" OR Milrinone is "Yes" OR Levosimendan is "Yes"}<br><br>(3) <u>Dose level of Norepinephrine plus Epinephrine</u>:<br>- None: Sum of Norepinephrine and Epinephrine dose =0<br>- Low: Sum of Norepinephrine and Epinephrine dose >0 AND ≤0.2 µg/kg/min<br>- Medium: Sum of Norepinephrine and Epinephrine dose >0.2 AND ≤0.4 µg/kg/min<br>- High: Sum of Norepinephrine and Epinephrine dose >0.4 µg/kg/min<br><br>0: Mean arterial pressure ≥70 mmHg AND Dopamine dose =0 AND Dobutamine dose =0 AND Epinephrine dose =0 AND Norepinephrine dose =0 AND Terlipressin is "No" AND Milrinone is "No" AND Levosimendan is "No"<br><br>1: Mean arterial pressure <70 mmHg AND Dopamine dose =0 AND Dobutamine dose =0 AND Epinephrine dose =0 AND Norepinephrine dose =0 AND Terlipressin is "No" AND Milrinone is "No" AND Levosimendan is "No"<br><br>2: [Dose level of Norepinephrine plus Epinephrine is "Low" AND Other vasopressor or inotrope is "No"] OR [Dose level of Norepinephrine plus Epinephrine is "None" AND Other vasopressor or inotrope is "Yes"]<br><br>3: [Dose level of Norepinephrine plus Epinephrine is "Medium" AND Other vasopressor or inotrope is "No"] OR [Dose level of Norepinephrine plus Epinephrine is "Low" AND Other vasopressor or inotrope is "Yes"]

	4: Dose level of Norepinephrine plus Epinephrine is "High" OR [Dose level of Norepinephrine plus Epinephrine is "Medium" AND Other vasopressor or inotrope is "Yes"]<br><br>We also checked instances where Dopamine was the sole vasopressor, and no further adjustments were needed beyond the previous steps.<br><br>Note that when the medications were recorded with a dose greater than zero, they were assumed to have been administered for at least one hour, given the absence of hour-level administration data. We assume that no vasoactive drugs were withheld due to a ceiling of treatment. Data on mechanical cardiovascular support are unavailable in the dataset.
Liver (original SOFA-1 from trial dataset)	10 (0.9%) values are missing.
Total serum bilirubin	10 values were missing; 13 records were converted from mg/dL to µmol/L by multiplying by 17.104. Records with values <1.7 or >800 µmol/L (n=6) were classified as missing. In total, 16 (1.5%) values are ultimately considered missing.
Liver (SOFA-1)	0: Total serum bilirubin <20 µmol/L or missing value<br>1: Total serum bilirubin <33 AND ≥20 µmol/L<br>2: Total serum bilirubin <102 AND ≥33 µmol/L<br>3: Total serum bilirubin ≤204 AND ≥102 µmol/L<br>4: Total serum bilirubin >204 µmol/L
Liver (SOFA-2)	0: Total serum bilirubin ≤20.6 µmol/L or missing<br>1: Total serum bilirubin ≤51.3 AND >20.6 µmol/L<br>2: Total serum bilirubin ≤102.6 AND >51.3 µmol/L<br>3: Total serum bilirubin ≤205 AND >102.6 µmol/L<br>4: Total serum bilirubin >205 µmol/L
Renal (original SOFA-1 from trial dataset)	7 (0.6%) values are missing.
Serum creatinine	8 values were missing; 5 records were converted from mg/dL to µmol/L by multiplying by 88.4. Records with values <8.8 or >999 µmol/L (n=2) were classified as missing. In total, 10 (0.9%) values are ultimately considered missing.
Urine output	Unit: mL/24h; 242 (22.2%) values are missing
Continuous renal replacement therapy	Yes vs. No; No missing value
Renal (SOFA-1)	#Score by serum creatinine<br>0: Serum creatinine <110 or missing<br>1: Serum creatinine <171 AND ≥110 µmol/L<br>2: Serum creatinine <300 AND ≥171 µmol/L<br>3: Serum creatinine ≤440 AND ≥300 µmol/L<br>4: Serum creatinine >440<br><br>#Score by urine output

	0: Urine output ≥500 mL/24h or missing<br>3: Urine output <500 AND ≥200 mL/24h<br>4: Urine output <200 mL/24h<br><br>The highest score (i.e., score by serum creatinine vs. by urine output) was then determined.
Kidney (SOFA-2)	Because data on urine output is needed in mL/kg/h, the variable weight was used to "convert" values from mL/24h to mL/kg/h. When weight is missing, it is imputed using age, sex, and height (when available) according to the following formulas: lm(Weight ~ Age + Sex + Height + I($Height^2$), data) OR lm(Weight ~ Age + Sex, data).<br><br>#Score by serum creatinine<br>0: Serum creatinine ≤110 µmol/L or missing<br>1: Serum creatinine ≤170 AND >110 µmol/L<br>2: Serum creatinine ≤300 AND >170 µmol/L<br>3: Serum creatinine >300 µmol/L<br><br>#Score by urine output<br>0: Urine output (mL/24h) is missing<br>2: Urine output (mL/24h)/Weight (kg)/24 ≥0.3 AND <1 mL/kg/h<br>3: Urine output (mL/24h)/Weight (kg)/24 <0.3 mL/kg/h<br><br>#Score by renal replacement therapy<br>0: Continuous renal replacement therapy is "No"<br>4: Continuous renal replacement therapy is "Yes"<br>Note that data on other types of renal replacement therapy are not available. We assumed no patients received renal replacement therapy exclusively for nonrenal causes. We also assumed there was no ceiling of treatment, machine unavailability, or decision to delay commencement in patients not receiving renal replacement therapy.<br><br>The highest score (i.e., score by serum creatinine, by urine output, and by renal replacement therapy) was then determined.
Coagulation (original SOFA-1 from trial dataset)	No missing value
Platelets	Unit: $\times 10^9$/L; No missing value; 1 (0.1%) record beyond 1-999 $\times 10^9$/L was classified as missing.
Coagulation (SOFA-1)	0: Platelets >150 $\times 10^9$/L or missing<br>1: Platelets >100 AND ≤150 $\times 10^9$/L<br>2: Platelets >50 AND ≤100 $\times 10^9$/L<br>3: Platelets >20 AND ≤50 $\times 10^9$/L<br>4: Platelets ≤20 $\times 10^9$/L

Hemostasis (SOFA-2)	0: Platelets >150 $\times 10^9$/L or missing<br>1: Platelets >100 AND ≤150 $\times 10^9$/L<br>2: Platelets >80 AND ≤100 $\times 10^9$/L<br>3: Platelets >50 AND ≤80 $\times 10^9$/L<br>4: Platelets ≤50 $\times 10^9$/L
Original SOFA-1 from trial dataset	No missing value
SOFA-1	Sum of Central nervous system (SOFA-1), Respiration (SOFA-1), Cardiovascular (SOFA-1), Liver (SOFA-1), Renal (SOFA-1), and Coagulation (SOFA-1)
SOFA-2	Sum of Central nervous system (SOFA-2), Respiratory (SOFA-2), Cardiovascular (SOFA-2), Liver (SOFA-2), Kidney (SOFA-2), and Hemostasis (SOFA-2)
Immune	Missing white blood cell was imputed as the median (i.e., 11.72$\times 10^9$/L).<br><br>0: [White blood cell ≥4 AND ≤10$\times 10^9$/L] AND Lymphocyte count >0.5$\times 10^9$/L<br>1: [{White blood cell ≥4 AND ≤10$\times 10^9$/L} AND Lymphocyte count ≤0.5$\times 10^9$/L] OR [White blood cell >10 AND ≤15$\times 10^9$/L]<br>2: [{White blood cell ≥1 AND <4$\times 10^9$/L} OR {White blood cell >15 AND ≤25$\times 10^9$/L}] & Lymphocyte count >0.5$\times 10^9$/L<br>3: [{White blood cell ≥1 AND <4$\times 10^9$/L} OR {White blood cell >15 AND ≤25$\times 10^9$/L}] & Lymphocyte count ≤0.5$\times 10^9$/L<br>4: White blood cell <1 OR >25$\times 10^9$/L
Clinical outcomes	
ICU mortality	1 (Yes) vs. 0 (No); No missing value
28-day mortality	1 (Yes) vs. 0 (No); No missing value
90-day mortality	1 (Yes) vs. 0 (No); No missing value
ICU-free days within 28 days	Unit: day; 1 (0.1%) are missing
Length of ICU stay within 28 days	Unit: day; 1 (0.1%) are missing
Length of ICU stay within 90 days	Unit: day; 1 (0.1%) are missing
Length of hospital stay	Unit: day; 1 (0.1%) are missing
Hospital readmission within 28 days	1 (Yes) vs. 0 (No); 6 (0.6%) are missing
New onset infection within 28 days	1 (Yes) vs. 0 (No); No missing value<br>Defined as an irrefutably positive culture (no identified pathogen) from an initially unaffected site or a new organism cultured from the original infected site; with either combined with clinician declaration of a definite infection based on clinical symptoms, laboratory tests results, and imaging
Pathogenic microorganism clearance within 28 days	1 (Yes) vs. 0 (No); 161 (14.8%) are missing<br>Defined as the transition of culture specimens from the previously definite pathogenic microorganisms to a negative state, coupled with the investigator's comprehensive assessment of clinical symptoms, laboratory tests results, and imaging
Mechanical ventilation within 28 days	1 (Yes) vs. 0 (No); No missing value

Mechanical ventilation-free days within 28 days	Unit: day; No missing value
Vasopressors within 28 days	1 (Yes) vs. 0 (No); No missing value
Vasopressors-free days within 28 days	Unit: day; No missing value
Continuous renal replacement therapy within 28 days	1 (Yes) vs. 0 (No); No missing value
Continuous renal replacement therapy-free days within 28 days	Unit: day; No missing value
SF-36 score (physical component) at day 90	537 (49.3%) are missing
SF-36 score (mental component) at day 90	537 (49.3%) are missing

Note: For simplicity, certain scoring rules are presented in abbreviated form. For example, a condition such as score =1 when $X \leq 10$; score =2 when $10 < X \leq 100$ may be shown simply as score = 2 when $X \leq 100$, with the lower bound implied by the preceding interval.

Abbreviations: SOFA, sequential organ failure assessment; ICU, intensive care unit; APACHE II, acute physiology and chronic health disease classification system II; FEU, fibrinogen equivalent units; PaO_2, partial pressure of arterial oxygen; FiO_2, fraction of inspired oxygen; SF-36, 36-item short form health survey.

2. R code for the primary analyses of adding immune markers to SOFA-2

```
rm(list=ls())
library(dplyr)
library(xgboost)
library(pROC)
library(ggplot2)
library(data.table)
set.seed(20251121)
################################################################################
vars <- c("WBC", "Lymphocyte_baseline", "mHLADR_baseline", "NLR_baseline", "Treg_baseline")

list_predictors <- unlist(
  lapply(1:length(vars), function(k) {
    combn(vars, k, simplify = FALSE)
  }),
  recursive = FALSE
)
rm(vars)
################################################################################
result <- data.frame(matrix(ncol=14,nrow=0))
colnames(result) <- c("Included_predictors",
                      "Best_par_objective",
                      "Best_par_eval_metric",
                      "Best_par_eta",
                      "Best_par_subsample",
                      "Best_par_colsample_bytree",
                      "Best_par_min_child_weight",
                      "Best_par_max_depth",
                      "Best_par_nrounds",
                      "AUC_SOFA2",
                      "AUC_SOFA2_predictors",
                      "AUC_diff",
                      "Dev_AUC_SOFA2",
                      "Dev_AUC_SOFA2_predictors")
################################################################################
for(abc in c(1:length(list_predictors))){
  print(abc)
  # Load data
  ##############################################################################
  dt <- readRDS("data_set_for_analysis.rds")
  scale_vars <- list_predictors[[abc]]
  dt <- dt[, c("DeathwithinICU","SOFA2", scale_vars)]
  ##############################################################################
  # 1. Hyperparameter tuning for XGBoost (5-fold CV on full data)
  ##############################################################################
  dt_tune <- dt
  for (v in scale_vars) {
    m <- mean(dt_tune[[v]], na.rm = TRUE)
    s <- sd(dt_tune[[v]], na.rm = TRUE)
    if (is.na(s) || s == 0) s <- 1
    dt_tune[[paste0(v, "_z")]] <- (dt_tune[[v]] - m) / s
  }
  rm(v,m,s)

  xgb_features_tune <- c("SOFA2", paste0(scale_vars, "_z"))

  dall_tune <- xgb.DMatrix(
    data  = as.matrix(dt_tune[, xgb_features_tune]),
    label = dt_tune$DeathwithinICU,
    missing = NA
  )
  rm(xgb_features_tune)

  # Hyperparameter grid
  max_depth_grid <- c(2, 3, 4, 5)
  nrounds_grid   <- c(50, 100, 200, 400)

  param_base <- list(
    objective        = "binary:logistic",
    eval_metric      = "auc",
    eta              = 0.05,
    subsample        = 0.8,
    colsample_bytree = 0.8,
    min_child_weight = 1
  )
```

```
cv_results <- list()

for (md in max_depth_grid) {
  for (nr in nrounds_grid) {
    params <- c(param_base, list(max_depth = md))

    cv <- xgb.cv(
      params     = params,
      data       = dall_tune,
      nrounds    = nr,
      nfold      = 5,
      stratified = TRUE,
      verbose    = FALSE,
      showsd     = TRUE,
      metrics    = "auc",
      prediction = FALSE
    )

    mean_auc  <- max(cv$evaluation_log$test_auc_mean)
    best_iter <- which.max(cv$evaluation_log$test_auc_mean)

    cv_results[[length(cv_results) + 1]] <- data.frame(
      max_depth     = md,
      nrounds       = nr,
      best_iter     = best_iter,
      test_auc_mean = mean_auc
    )
    rm(params,cv,mean_auc,best_iter)
  }
  rm(nr)
}
rm(md,max_depth_grid,nrounds_grid)

cv_table <- rbindlist(cv_results)
best_row <- cv_table[which.max(test_auc_mean)]
#print(best_row)

best_max_depth <- best_row$max_depth
best_nrounds   <- best_row$best_iter

best_params <- c(
  param_base,
  list(max_depth = best_max_depth)
)
################################################################################
#Developed (apparent) AUC for models fitted on the full data
#Recreate z-scored predictors on FULL data for the final model
dt_dev <- dt
for (v in scale_vars) {
  m <- mean(dt_dev[[v]], na.rm = TRUE)
  s <- sd(dt_dev[[v]], na.rm = TRUE)
  if (is.na(s) || s == 0) s <- 1
  dt_dev[[paste0(v, "_z")]] <- (dt_dev[[v]] - m) / s
}
rm(v,m,s)

# Features for the xgboost model
xgb_features_dev <- c("SOFA2", paste0(scale_vars, "_z"))

##Developed SOFA2-only logistic (full data)
complete_dev_logit <- dt_dev[complete.cases(dt_dev[, c("DeathwithinICU", "SOFA2")]), ]

dev_logit_model <- glm(DeathwithinICU ~ SOFA2,
                       data   = complete_dev_logit,
                       family = binomial)

dev_logit_pred <- predict(dev_logit_model,
                          newdata = complete_dev_logit,
                          type    = "response")

roc_dev_logit <- roc(response  = complete_dev_logit$DeathwithinICU,
                     predictor = dev_logit_pred,
                     quiet     = TRUE)
```

```
dev_auc_logit <- as.numeric(auc(roc_dev_logit))
dev_ci_logit  <- as.numeric(ci.auc(roc_dev_logit, conf.level = 0.95))

#Developed XGBoost model (SOFA2 + markers; full data)
d_dev <- xgb.DMatrix(
  data   = as.matrix(dt_dev[, xgb_features_dev]),
  label  = dt_dev$DeathwithinICU,
  missing = NA
)

xgb_model_dev <- xgb.train(
  params  = best_params,
  data    = d_dev,
  nrounds = best_nrounds,
  verbose = FALSE
)

dev_xgb_pred <- predict(xgb_model_dev, d_dev)

roc_dev_xgb <- roc(response  = dt_dev$DeathwithinICU,
                   predictor = dev_xgb_pred,
                   quiet     = TRUE)

dev_auc_xgb <- as.numeric(auc(roc_dev_xgb))
dev_ci_xgb  <- as.numeric(ci.auc(roc_dev_xgb, conf.level = 0.95))

rm(dt_dev, xgb_features_dev, complete_dev_logit,
   dev_logit_model, dev_logit_pred, roc_dev_logit,
   d_dev, xgb_model_dev, dev_xgb_pred, roc_dev_xgb)
################################################################################
rm(dt_tune, dall_tune, cv_results, cv_table, best_row, best_max_depth,param_base)
################################################################################
# 2. Bootstrap evaluation of AUCs (SOFA2-logit vs XGBoost)
################################################################################
B <- 2000  # number of bootstrap resamples

auc_logit_vec <- rep(NA_real_, B)
auc_xgb_vec   <- rep(NA_real_, B)
diff_vec      <- rep(NA_real_, B)

n <- nrow(dt)

for (b in 1:B) {
  #cat("Bootstrap replicate:", b, "of", B, "\n")

  #-----------------------------
  # 2.1. Bootstrap sample indices
  #-----------------------------
  idx_boot <- sample(seq_len(n), replace = TRUE)
  idx_oob  <- setdiff(seq_len(n), unique(idx_boot))

  # Need some out-of-bag observations
  if (length(idx_oob) < 30) next

  dt_train <- dt[idx_boot, ]
  dt_test  <- dt[idx_oob, ]

  #-----------------------------
  # 2.2. Z-score scaling using TRAIN only
  #-----------------------------
  for (v in scale_vars) {
    m <- mean(dt_train[[v]], na.rm = TRUE)
    s <- sd(dt_train[[v]], na.rm = TRUE)
    if (is.na(s) || s == 0) s <- 1  # avoid division by zero

    dt_train[[paste0(v, "_z")]] <- (dt_train[[v]] - m) / s
    dt_test[[paste0(v, "_z")]]  <- (dt_test[[v]]  - m) / s
  }
  rm(v, m, s)

  xgb_features <- c("SOFA2", paste0(scale_vars, "_z"))

  #-----------------------------
  # 2.3. Baseline model: SOFA2-only logistic
  #-----------------------------
```

```
complete_train_logit <- dt_train[complete.cases(dt_train[, c("DeathwithinICU", "SOFA2")]), ]
complete_test_logit  <- dt_test[complete.cases(dt_test[, c("DeathwithinICU", "SOFA2")]), ]

if (nrow(complete_train_logit) < 30 || nrow(complete_test_logit) < 30) next

logit_model <- try(
  glm(DeathwithinICU ~ SOFA2,
      data   = complete_train_logit,
      family = binomial),
  silent = TRUE
)
if (inherits(logit_model, "try-error")) next

logit_pred <- try(
  predict(logit_model,
          newdata = complete_test_logit,
          type    = "response"),
  silent = TRUE
)
if (inherits(logit_pred, "try-error")) next

roc_logit <- try(
  roc(response  = complete_test_logit$DeathwithinICU,
      predictor = logit_pred,
      quiet     = TRUE),
  silent = TRUE
)
if (inherits(roc_logit, "try-error")) next

auc_logit <- as.numeric(auc(roc_logit))

#----------------------------
# 2.4. XGBoost model: SOFA2 + z-scored predictors
#----------------------------
dtrain <- xgb.DMatrix(
  data   = as.matrix(dt_train[, xgb_features]),
  label  = dt_train$DeathwithinICU,
  missing = NA
)

dtest <- xgb.DMatrix(
  data   = as.matrix(dt_test[, xgb_features]),
  label  = dt_test$DeathwithinICU,
  missing = NA
)

xgb_model <- try(
  xgb.train(
    params  = best_params,
    data    = dtrain,
    nrounds = best_nrounds,
    verbose = FALSE
  ),
  silent = TRUE
)
if (inherits(xgb_model, "try-error")) next

xgb_pred <- try(
  predict(xgb_model, dtest),
  silent = TRUE
)
if (inherits(xgb_pred, "try-error")) next

roc_xgb <- try(
  roc(response  = dt_test$DeathwithinICU,
      predictor = xgb_pred,
      quiet     = TRUE),
  silent = TRUE
)
if (inherits(roc_xgb, "try-error")) next

auc_xgb <- as.numeric(auc(roc_xgb))

#----------------------------
# 2.5. Store results
```

```
    #-----------------------------
    auc_logit_vec[b] <- auc_logit
    auc_xgb_vec[b]   <- auc_xgb
    diff_vec[b]      <- auc_xgb - auc_logit

    rm(idx_boot,idx_oob,dt_train,dt_test,xgb_features,complete_train_logit,
       complete_test_logit,logit_model,logit_pred,roc_logit,auc_logit,dtrain,
       dtest,xgb_model,xgb_pred,roc_xgb,auc_xgb)

  }
  rm(b, B, n)
  ##############################################################################
  # 3. Summarize bootstrap results
  ##############################################################################
  valid <- !is.na(diff_vec)

  mean_auc_logit <- mean(auc_logit_vec[valid])
  mean_auc_xgb   <- mean(auc_xgb_vec[valid])
  mean_diff      <- mean(diff_vec[valid])

  ci_logit <- quantile(auc_logit_vec[valid], probs = c(0.025, 0.975))
  ci_xgb   <- quantile(auc_xgb_vec[valid],   probs = c(0.025, 0.975))
  ci_diff  <- quantile(diff_vec[valid],      probs = c(0.025, 0.975))
  rm(auc_logit_vec, auc_xgb_vec, valid, diff_vec)
  ##############################################################################
  result_temp <- data.frame(Included_predictors=paste0(list_predictors[abc], collapse=", "),
                            Best_par_objective=best_params$objective,
                            Best_par_eval_metric=best_params$eval_metric,
                            Best_par_eta=best_params$eta,
                            Best_par_subsample=best_params$subsample,
                            Best_par_colsample_bytree=best_params$colsample_bytree,
                            Best_par_min_child_weight=best_params$min_child_weight,
                            Best_par_max_depth=best_params$max_depth,
                            Best_par_nrounds=best_nrounds,
                            AUC_SOFA2=paste0(trimws(format(round(mean_auc_logit, digits=3), nsmall=3))," (",
                                      trimws(format(round(ci_logit[1], digits=3), nsmall=3)),"-",
                                      trimws(format(round(ci_logit[2], digits=3), nsmall=3)),")"),
                            AUC_SOFA2_predictors=paste0(trimws(format(round(mean_auc_xgb, digits=3), nsmall=3))," (",
                                      trimws(format(round(ci_xgb[1], digits=3), nsmall=3)),"-",
                                      trimws(format(round(ci_xgb[2], digits=3), nsmall=3)),")"),
                            AUC_diff=paste0(trimws(format(round(mean_diff, digits=3), nsmall=3))," (",
                                      trimws(format(round(ci_diff[1], digits=3), nsmall=3))," to ",
                                      trimws(format(round(ci_diff[2], digits=3), nsmall=3)),")"),
                            Dev_AUC_SOFA2=paste0(trimws(format(round(dev_auc_logit, digits=3), nsmall=3))," (",
                                      trimws(format(round(dev_ci_logit[1], digits=3), nsmall=3)),"-",
                                      trimws(format(round(dev_ci_logit[3], digits=3), nsmall=3)),")"),
                            Dev_AUC_SOFA2_predictors=paste0(trimws(format(round(dev_auc_xgb, digits=3), nsmall=3))," (",
                                      trimws(format(round(dev_ci_xgb[1], digits=3), nsmall=3)),"-",
                                      trimws(format(round(dev_ci_xgb[3], digits=3), nsmall=3)),")"))
  result <- rbind(result, result_temp)
  rm(result_temp)
  ##############################################################################
  rm(dt, best_params, best_nrounds, mean_auc_logit, mean_auc_xgb, mean_diff,
     ci_diff, ci_xgb, ci_logit, scale_vars, dev_auc_logit, dev_auc_xgb,
     dev_ci_logit, dev_ci_xgb)
}
rm(list_predictors, abc)
##############################################################################
result <- result[, c("Included_predictors",
                     "Dev_AUC_SOFA2",
                     "AUC_SOFA2",
                     "Dev_AUC_SOFA2_predictors",
                     "AUC_SOFA2_predictors",
                     "AUC_diff",
                     "Best_par_objective",
                     "Best_par_eval_metric",
                     "Best_par_eta",
                     "Best_par_subsample",
                     "Best_par_colsample_bytree",
                     "Best_par_min_child_weight",
                     "Best_par_max_depth",
                     "Best_par_nrounds")]

xlsx::write.xlsx(result, "AUCs after adding immune markers.xlsx", row.names=F)
rm(result)
```

e-Tables

e-Table 1. Baseline characteristics and clinical outcomes of the study participants

**Variable**	**Summary statistics**
n	1089
Age (years)	64.5 (51.5-73.1)
<60	427 (39.2)
≥60	662 (60.8)
Sex	
Male	750 (68.9)
Female	339 (31.1)
Height (cm)	168.0 (160.0-172.0)
Weight (kg)	64.0 (54.8-74.1)
Admission to ICU after a surgical procedure	538 (49.4)
Pre-existing conditions	
Hypertension	421 (38.7)
Coronary heart disease	128 (11.8)
Chronic obstructive pulmonary disease	88 (8.1)
Diabetes mellitus	277 (25.4)
Chronic kidney disease	64 (5.9)
Solid malignant tumors	121 (11.1)
Infection sites	
Lung	343 (31.5)
Abdomen	108 (9.9)
Bloodstream	86 (7.9)
Urinary tract	24 (2.2)
Biliary tract	26 (2.4)
Skin and soft tissue	9 (0.8)
Others	42 (3.9)
Multiple sites	216 (19.8)
Unknown	231 (21.2)
Missing	4 (0.4)
Microorganisms	
Gram negative	379 (34.8)
Gram positive	77 (7.1)
Fungi	100 (9.2)
Atypical pathogens	3 (0.3)
Mixed	293 (26.9)
Culture negative	233 (21.4)
Missing	4 (0.4)
APACHE II score	14 (10-19)
Immune markers	
White blood cell ($\times 10^9$/L)	11.7 (7.7-16.7)
Lymphocyte count ($\times 10^9$/L)	0.7 (0.4-1.1)
Monocyte human leucocyte antigen-DR (antibodies per cell)	4185.0 (2415.5-7184.5)
Neutrophil-to-lymphocyte ratio	13.9 (8.0-23.4)
Regulatory T cells (%)	9.0 (7.0-11.6)
Other laboratory results	
Neutrophil count ($\times 10^9$/L)	9.8 (6.1-14.7)
C reactive protein (mg/L)	127.8 (70.8-188.7)
Procalcitonin (ng/mL)	3.3 (0.8-22.0)
Lactate (mmol/L)	1.7 (1.2-2.4)
Prothrombin time (s)	15.1 (13.6-16.8)
Activated partial thromboplastin time (s)	38.9 (33.0-46.5)
International normalized ratio	1.3 (1.1-1.4)
D-dimer (ng/mL FEU)	4.6 (2.5-8.9)
Treatment arm assigned in the original trial	
Placebo	547 (50.2)
Thymosin alpha1	542 (49.8)

Clinical outcomes	
ICU mortality	100 (9.2)
28-day mortality	259 (23.8)
90-day mortality	345 (31.7)
ICU-free days within 28 days (days)	13.0 (0.0-20.0)
Length of ICU stay within 28 days (days)	15.0 (8.0-28.0)
Length of ICU stay within 90 days (days)	15.0 (8.0-30.0)
Length of hospital stay (days)	23.0 (14.0-28.0)
Hospital readmission within 28 days	147 (13.6)
New onset infection within 28 days	280 (25.7)
Pathogenic microorganism clearance within 28 days	170 (18.3)
Mechanical ventilation within 28 days	599 (55.0)
Mechanical ventilation-free days within 28 days (days)	25.0 (9.0-28.0)
Vasopressors within 28 days	454 (41.7)
Vasopressors-free days within 28 days (days)	27.0 (15.0-28.0)
Continuous renal replacement therapy within 28 days	147 (13.5)
Continuous renal replacement therapy-free days within 28 days (days)	28.0 (20.0-28.0)
SF-36 score (physical component) at day 90	61.1 (52.8-68.0)
SF-36 score (mental component) at day 90	61.5 (51.8-70.1)

Note: Continuous variables are summarized as median (25th-75th percentile), and categorical variables as frequency (percentage). Missing values were excluded on a per-variable basis (except for variables treated as categorical). Please refer to e-Methods for the number of missing observations for each variable.
Abbreviations: ICU, intensive care unit; FEU, Fibrinogen Equivalent Units; SF-36, 36-item short form health survey.

e-Table 2. Summary statistics about baseline characteristics involved in SOFA score calculation

	Original SOFA-1 from trial dataset	SOFA-1	SOFA-2
**Total**	7 (5-10)	7 (5-10)	6 (4-9)
**Central nervous system/Brain**	0 (0-2)	0 (0-2)	0 (0-2)
Glasgow Coma Scale	15 (11-15)		
Drugs to treat delirium	1 (0.1)		
**Respiration/Respiratory**	2 (1-3)	2 (1-3)	1 (0-2)
PaO_2 (mmHg)	100.0 (79.6-128.0)		
FiO_2	0.4 (0.4-0.5)		
PaO_2: FiO_2 ratio (mmHg)	227.5 (154.0-305.5)		
Mechanical ventilation	836 (76.8)		
**Cardiovascular**	2 (0-4)	1 (0-4)	1 (0-2)
Mean arterial pressure (mmHg)	85.3 (75.3-96.0)		
Norepinephrine dose (µg/kg/min)	0.0 (0.0-0.2)		
Epinephrine dose (µg/kg/min)	0.0 (0.0-0.0)		
Dopamine dose (µg/kg/min)	0.0 (0.0-0.0)		
Dobutamine dose (µg/kg/min)	0.0 (0.0-0.0)		
Terlipressin	3 (0.3)		
Milrinone	7 (0.6)		
Levosimendan	7 (0.6)		
**Liver**	0 (0-2)	0 (0-2)	0 (0-1)
Total serum bilirubin (µmol/L)	19.2 (12.1-37.5)		
**Renal/Kidney**	0 (0-2)	0 (0-2)	1 (0-2)
Serum creatinine (µmol/L)	89.2 (58.0-155.3)		
Urine output (mL/24h)	2000.0 (1182.5-2940.0)		
Continuous renal replacement therapy	184 (16.9)		
**Coagulation/Hemostasis**	0 (0-2)	0 (0-2)	0 (0-2)
Platelets ($\times 10^9$/L)	153.0 (85.0-234.2)		

Note: Continuous variables are summarized as median (25th-75th percentile), and categorical variables as frequency (percentage). Missing values were excluded on a per-variable basis. Please refer to e-Methods for the number of missing observations for each variable.
Abbreviations: SOFA, sequential organ failure assessment; PaO_2, partial pressure of arterial oxygen; FiO_2, fraction of inspired oxygen.

e-Table 3. Comparison of SOFA-1 and SOFA-2 scores

Variable	SOFA-1	SOFA-2	p-value	SMD
n	1089	1089		
Total				
Median (IQR)	7 (5-10)	6 (4-9)	0.001	0.120
Category			0.005	0.154
≤5	356 (32.7)	432 (39.7)		
6-10	503 (46.2)	455 (41.8)		
11-15	194 (17.8)	162 (14.9)		
>15	36 (3.3)	40 (3.7)		
Central nervous system/Brain				
Median (IQR)	0 (0-2)	0 (0-2)	0.801	0.019
Category			0.200	0.105
0	610 (56.0)	609 (55.9)		
1	133 (12.2)	134 (12.3)		
2	135 (12.4)	163 (15.0)		
3	131 (12.0)	103 (9.5)		
4	80 (7.3)	80 (7.3)		
Respiration/Respiratory				
Median (IQR)	2 (1-3)	1 (0-2)	<0.001	0.501
Category			<0.001	0.574
0	93 (8.5)	290 (26.6)		
1	199 (18.3)	266 (24.4)		
2	431 (39.6)	303 (27.8)		
3	260 (23.9)	153 (14.0)		
4	106 (9.7)	77 (7.1)		
Cardiovascular				
Median (IQR)	1 (0-4)	1 (0-2)	<0.001	0.262
Category			<0.001	0.869
0	526 (48.3)	519 (47.7)		
1	51 (4.7)	51 (4.7)		
2	16 (1.5)	273 (25.1)		
3	151 (13.9)	132 (12.1)		
4	345 (31.7)	114 (10.5)		
Liver				
Median (IQR)	0 (0-2)	0 (0-1)	0.017	0.120
Category			<0.001	0.363
0	576 (52.9)	592 (54.4)		
1	204 (18.7)	310 (28.5)		
2	215 (19.7)	94 (8.6)		
3	60 (5.5)	59 (5.4)		
4	34 (3.1)	34 (3.1)		
Renal/Kidney				
Median (IQR)	0 (0-2)	1 (0-2)	<0.001	0.356
Category			<0.001	0.432
0	646 (59.3)	492 (45.2)		
1	161 (14.8)	111 (10.2)		
2	128 (11.8)	240 (22.0)		
3	65 (6.0)	62 (5.7)		
4	89 (8.2)	184 (16.9)		
Coagulation/Hemostasis				
Median (IQR)	0 (0-2)	0 (0-2)	0.082	0.148
Category			<0.001	0.451
0	552 (50.7)	552 (50.7)		
1	204 (18.7)	204 (18.7)		
2	209 (19.2)	84 (7.7)		
3	85 (7.8)	125 (11.5)		
4	39 (3.6)	124 (11.4)		

Abbreviations: SOFA, sequential organ failure assessment; SMD, standardized mean difference; IQR, interquartile range.

e-Table 4. Baseline characteristics of the study participants by level of SOFA score

Variable	SOFA-1≥2<br>SOFA-2≥2	SOFA-1≥2<br>SOFA-2<2	SOFA-1<2<br>SOFA-2<2
n	1054 (96.8)	24 (2.2)	11 (1.0)
Age (years)	65.0 (52.3-73.4)	43.3 (36.0-56.1)	39.7 (33.0-47.9)
<60	398 (37.8)	19 (79.2)	10 (90.9)
≥60	656 (62.2)	5 (20.8)	1 (9.1)
Sex			
Male	726 (68.9)	19 (79.2)	5 (45.5)
Female	328 (31.1)	5 (20.8)	6 (54.5)
Height (cm)	168.0 (160.0-172.0)	169.0 (162.2-172.0)	170.0 (170.0-176.0)
Weight (kg)	63.5 (54.0-72.2)	70.0 (55.0-82.0)	75.0 (68.0-76.0)
Admission to ICU after a surgical procedure	520 (49.3)	13 (54.2)	5 (45.5)
Pre-existing conditions			
Hypertension	414 (39.3)	6 (25.0)	1 (9.1)
Coronary heart disease	127 (12.0)	1 (4.2)	0 (0.0)
Chronic obstructive pulmonary disease	86 (8.2)	2 (8.3)	0 (0.0)
Diabetes mellitus	269 (25.5)	6 (25.0)	2 (18.2)
Chronic kidney disease	61 (5.8)	2 (8.3)	1 (9.1)
Solid malignant tumors	121 (11.5)	0 (0.0)	0 (0.0)
Infection sites			
Lung	337 (32.0)	4 (16.7)	2 (18.2)
Abdomen	103 (9.8)	3 (12.5)	2 (18.2)
Bloodstream	81 (7.7)	3 (12.5)	2 (18.2)
Urinary tract	24 (2.3)	0 (0.0)	0 (0.0)
Biliary tract	23 (2.2)	3 (12.5)	0 (0.0)
Skin and soft tissue	8 (0.8)	1 (4.2)	0 (0.0)
Others	38 (3.6)	2 (8.3)	2 (18.2)
Multiple sites	210 (19.9)	4 (16.7)	2 (18.2)
Unknown	226 (21.4)	4 (16.7)	1 (9.1)
Missing	4 (0.4)	0 (0.0)	0 (0.0)
Microorganisms			
Gram negative	364 (34.5)	7 (29.2)	8 (72.7)
Gram positive	75 (7.1)	2 (8.3)	0 (0.0)
Fungi	98 (9.3)	2 (8.3)	0 (0.0)
Atypical pathogens	3 (0.3)	0 (0.0)	0 (0.0)
Mixed	282 (26.8)	9 (37.5)	2 (18.2)

Culture negative	228 (21.6)	4 (16.7)	1 (9.1)
Missing	4 (0.4)	0 (0.0)	0 (0.0)
APACHE II score	15 (10-19)	8.5 (6.8-11)	6 (5.5-8.5)
Immune markers			
White blood cell ($\times10^9$/L)	11.8 (7.7-16.8)	9.7 (6.7-14.0)	9.5 (8.3-11.9)
Lymphocyte count ($\times10^9$/L)	0.7 (0.4-1.1)	0.9 (0.7-1.2)	0.8 (0.7-1.1)
Monocyte human leucocyte antigen-DR (antibodies per cell)	4059.0 (2398.5-6868.1)	9439.0 (4564.9-12269.0)	11845.9 (7044.4-14854.3)
Neutrophil-to-lymphocyte ratio	14.0 (8.1-23.7)	8.9 (5.5-17.1)	12.5 (7.4-13.5)
Regulatory T cells (%)	9.0 (7.0-11.5)	8.3 (5.6-11.7)	10.2 (3.0-12.3)
Other laboratory results			
Neutrophil count ($\times10^9$/L)	9.9 (6.1-14.8)	8.2 (4.6-12.4)	8.2 (6.8-10.5)
C reactive protein (mg/L)	127.8 (70.8-188.5)	154.4 (66.6-199.6)	95.4 (59.3-152.9)
Procalcitonin (ng/mL)	3.4 (0.8-22.4)	0.8 (0.2-1.7)	0.4 (0.1-0.6)
Lactate (mmol/L)	1.7 (1.2-2.4)	1.5 (1.3-1.5)	0.7 (0.7-0.7)
Prothrombin time (s)	15.1 (13.6-16.8)	15.6 (14.6-17.7)	14.2 (12.5-16.5)
Activated partial thromboplastin time (s)	39.0 (33.1-46.5)	38.8 (31.1-44.5)	36.2 (27.9-38.0)
International normalized ratio	1.3 (1.1-1.4)	1.4 (1.3-1.5)	1.2 (1.1-1.4)
D-dimer (ng/mL FEU)	4.6 (2.5-9.0)	4.2 (3.0-6.2)	2.4 (1.6-4.4)
Treatment arm assigned in the original trial			
Placebo	526 (49.9)	14 (58.3)	7 (63.6)
Thymosin alpha1	528 (50.1)	10 (41.7)	4 (36.4)

Note: Continuous variables are summarized as median (25th-75th percentile), and categorical variables as frequency (percentage). Missing values were excluded on a per-variable basis (except for variables treated as categorical). Please refer to e-Methods for the number of missing observations for each variable.
Abbreviations: SOFA, sequential organ failure assessment; ICU, intensive care unit; FEU, Fibrinogen Equivalent Units.

e-Table 5. Clinical outcomes of the study participants by level of SOFA score

Variable	**SOFA-1≥2 SOFA-2≥2**	**SOFA-1≥2 SOFA-2<2**	**SOFA-1<2 SOFA-2<2**
n	1054 (96.8)	24 (2.2)	11 (1.0)
ICU mortality	100 (9.5)	0 (0.0)	0 (0.0)
28-day mortality	256 (24.3)	2 (8.3)	1 (9.1)
90-day mortality	340 (32.3)	3 (12.5)	2 (18.2)
ICU-free days within 28 days (days)	14.0 (0.0-20.0)	0.0 (0.0-8.2)	0.0 (0.0-3.0)
Length of ICU stay within 28 days (days)	14.0 (8.0-28.0)	28.0 (19.8-28.0)	28.0 (25.0-28.0)
Length of ICU stay within 90 days (days)	14.0 (8.0-29.0)	31.0 (19.8-59.5)	40.0 (30.0-50.5)
Length of hospital stay (days)	23.0 (13.0-28.0)	28.0 (22.5-28.0)	28.0 (28.0-28.0)
Hospital readmission within 28 days	141 (13.5)	4 (16.7)	2 (18.2)
New onset infection within 28 days	272 (25.8)	5 (20.8)	3 (27.3)
Pathogenic microorganism clearance within 28 days	166 (18.5)	4 (20.0)	0 (0.0)
Mechanical ventilation within 28 days	586 (55.6)	9 (37.5)	4 (36.4)
Mechanical ventilation-free days within 28 days (days)	25.0 (9.0-28.0)	28.0 (16.2-28.0)	28.0 (22.0-28.0)
Vasopressors within 28 days	442 (41.9)	8 (33.3)	4 (36.4)
Vasopressors-free days within 28 days (days)	27.0 (15.0-28.0)	28.0 (25.0-28.0)	28.0 (17.5-28.0)
Continuous renal replacement therapy within 28 days	147 (13.9)	0 (0.0)	0 (0.0)
Continuous renal replacement therapy-free days within 28 days (days)	28.0 (20.0-28.0)	28.0 (28.0-28.0)	28.0 (28.0-28.0)
SF-36 score (physical component) at day 90	61.0 (52.8-68.0)	68.0 (51.9-85.2)	66.0 (58.9-77.2)
SF-36 score (mental component) at day 90	61.0 (51.8-70.1)	66.5 (58.6-68.1)	68.5 (64.9-72.7)

Note: Missing values were excluded on a per-variable basis. Please refer to e-Methods for the number of missing observations for each variable.
Abbreviations: SOFA, sequential organ failure assessment; ICU, intensive care unit; IQR, interquartile range; SF-36, 36-item short form health survey.

e-Figure

e-Figure 1. Discriminatory power for mortality between SOFA-2 and SOFA-2 augmented with the immune domain

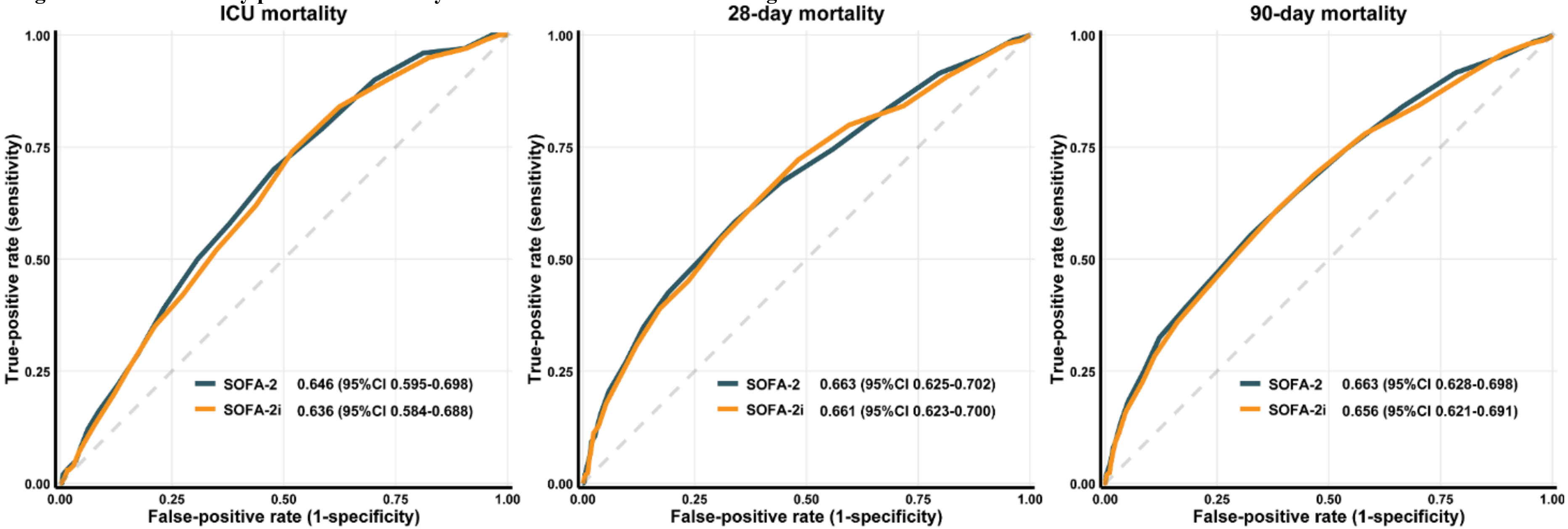

Note: The SOFA-2i refers to the SOFA-2 score with the additional immune domain proposed in the original study that developed the SOFA-2 score.
Abbreviations: SOFA, sequential organ failure assessment; ICU, intensive care unit; CI, confidence interval.
